# Tetramethylpyrazine alleviates acute pancreatitis inflammation by inhibiting pyroptosis via the NRF2 pathway

**DOI:** 10.3389/fphar.2025.1557681

**Published:** 2025-04-23

**Authors:** Huangen Li, Yi Gao, Minglian Huang, Hongling Zhang, Qingqing Wu, Youpei Huang, Xiaotong Ye, Weiwen Chen

**Affiliations:** ^1^ Department of Critical Care Medicine, Quanzhou First Hospital Affiliated to Fujian Medical University, Quanzhou, China; ^2^ Department of General Medicine, Xiamen Changgeng Hospital Affiliated to Huaqiao University, Xiamen, China; ^3^ School of Medicine, Huaqiao University, Quanzhou, China

**Keywords:** active alkaloid, anti-inflammatory, anti-pyroptotic, NLRP3 inflammasome, nuclear factor erythroid 2-related factor 2

## Abstract

**Objective:**

Tetramethylpyrazine (TMPZ), an active alkaloid derived from traditional Chinese medicine, has shown anti-inflammatory and anti-pyroptotic properties. However, its role in acute pancreatitis (AP)-induced pyroptosis remains unclear. This study aims to investigate the effects of TMPZ on AP-induced pyroptosis and its potential mechanisms.

**Materials and methods:**

A cerulein-induced AP rat model was used to evaluate TMPZ’s protective effects *in vivo*, and its mechanisms were explored using AR42J cells *in vitro*. Pancreatic injury was assessed by hematoxylin-eosin staining, TUNEL assay, and serum biochemistry. Transmission electron microscopy, immunofluorescence, Western blotting, and quantitative real-time polymerase chain reaction (RT-qPCR) were conducted to examine pyroptosis and related signaling pathways. Cytotoxicity and apoptosis were measured by CCK-8, LDH assays, and Hoechst 33342/PI staining. The role of NRF2 in TMPZ’s effects was further evaluated using NRF2 siRNA.

**Results:**

TMPZ alleviated pancreatic histopathological damage, reduced apoptosis, and decreased serum amylase levels and pro-inflammatory cytokines (IL-1β, IL-18). TMPZ also suppressed pyroptosis by inhibiting NLRP3 inflammasome activation and downregulating pyroptosis-related proteins (NLRP3,caspase-1, ASC, GSDMD) while upregulating NRF2 and HO-1 expression. NRF2 siRNA attenuated TMPZ’s anti-inflammatory and pyroptosis-inhibitory effects, confirming the involvement of the NRF2 pathway.

**Conclusion:**

TMPZ mitigates AP-induced inflammation and injury by modulating pyroptosis via the NRF2 signaling pathway. These findings suggest TMPZ’s therapeutic potential for AP.

## Graphical Abstract

**Figure:**
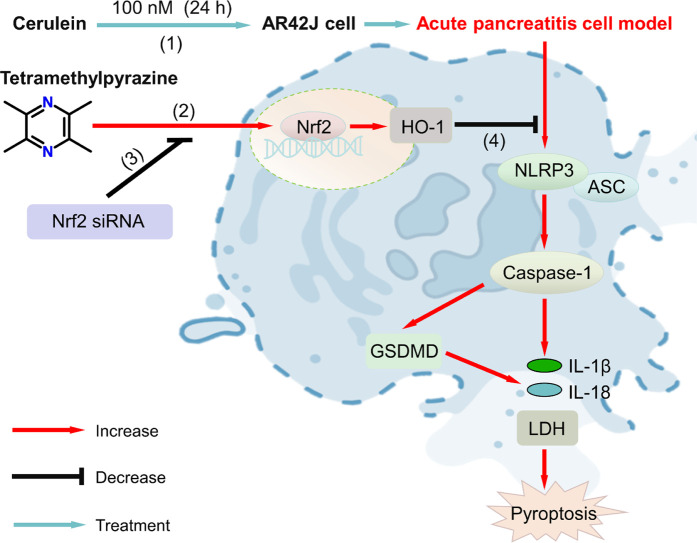
Proposed signaling pathway of tetramethylpyrazine (TMPZ) in protecting against acute pancreatitis through inhibition of pyroptosis via the NRF2 pathway. (1) AP cell model was established by treating AR42J cells with 100 nM cerulein for 24 h; (2) TMPZ promoted translocation of NRF2 protein from the cytoplasm to the nucleus and activated HO-1 protein expression; (3) NRF2 siRNA inhibited the effect of TMPZ; (4) TMPZ reduced pyroptosis in AP cells by modulating levels of IL-1β, IL-18, and expression of NLRP3, caspase-1, ASC, and GSDMD at both mRNA and protein levels.

## 1 Introduction

Acute pancreatitis (AP) is a common digestive system disease characterized by acute onset, rapid progression, and severe clinical course (Horwitz and Birk, 2024). The global incidence is approximately 13–45 cases per 100,000 people annually (Mederos et al., 2021), with an increasing trend (Petrov and Yadav, 2019). About 20%–30% of AP patients develop severe acute pancreatitis (SAP) and/or necrotizing pancreatitis, which pose significant treatment challenges, incur high costs, and have poor prognoses, with mortality rates of 36%–50% (Portelli and Jones, 2017; Gao et al., 2021). AP is primarily marked by aberrant activation of pancreatic enzymes, leading to injury of pancreatic tissues and surrounding organs. The pathogenesis is complex, and the pathophysiological mechanisms remain incompletely understood.

Pyroptosis, a newly discovered pro-inflammatory programmed cell death distinct from apoptosis, has been implicated in the progression of AP (Lu et al., 2023; Cao et al., 2024; Feng et al., 2024; Liu et al., 2024). Pyroptosis is a caspase-dependent and highly immunogenic form of cell death involving inflammasome NLRP3 activation, caspase-dependent cleavage of gasdermin D (GSDMD), apoptosis-associated speck-like protein (ASC), and pro-inflammatory cytokine release (Kayagaki et al., 2015; Xue et al., 2019; Lu et al., 2023). Recent studies have found that inhibiting pyroptotic pathways in pancreatic acinar cells, such as NLRP3 and GSDMD, significantly reduces inflammation and pancreatic injury (Gao et al., 2021; Lu et al., 2023; Wang et al., 2023; Lyu et al., 2024). Pyroptosis has been confirmed as the primary cell death type in AP acinar cells, triggering local and systemic inflammatory responses (Gao et al., 2021; Al Mamun et al., 2022). Thus, inhibition of the pyroptosis pathway may hold substantial therapeutic potential for AP treatment.

The nuclear factor erythroid 2–related factor 2 (NRF2) pathway has been implicated in pyroptosis (Niu et al., 2024). NRF2 is expressed in the liver, kidneys, spleen, and heart, where it is primarily localized in the cytoplasm (He et al., 2020; Kong et al., 2021a; Niu et al., 2024). In response to oxidative stress, NRF2 dissociates from Keap1, translocates from the cytoplasm to the nucleus, and induces the expression of HO-1 (Zimmermann et al., 2015). In the absence or knockout of NRF2, pancreatic cells become more susceptible to damage by ROS, inflammatory mediators, and toxic metabolites (Yuan et al., 2023). The reduction in oxidative stress and inflammation in pancreatic acinar cells is linked to the activation of the NRF2/HO-1 signaling pathway. Activation of this pathway reduces NLRP3 production and inflammatory cytokine release, ultimately improving AP and related organ damage (Mei et al., 2020; Kong et al., 2021b), indicating that the NRF2/HO-1 pathway is a critical protective mechanism in AP.

In recent years, traditional Chinese herbal medicine has been widely applied to treat AP and is considered a significant non-surgical treatment option, with its efficacy validated through both clinical practice and basic research (Chen et al., 2024; Deng et al., 2024; Li et al., 2024). Tetramethylpyrazine (TMPZ), an active alkaloid monomer primarily purified from the tubers of herbs such as Curcuma, Jatropha, and Ligustrum, possesses a defined chemical structure and exhibits multiple therapeutic effects (Qi et al., 2024), including anti-inflammatory action, reactive oxygen species scavenging, lipid peroxidation inhibition, microcirculatory improvement, and immunomodulation. Chen et al. (2016) found that TMPZ alleviates AP by promoting early acinar cell apoptosis through inhibition of the p38 and Erk MAPK pathways. Recent studies have reported that TMPZ mitigates pulmonary toxicity (Li et al., 2023) and coronary artery calcification (Yang et al., 2024) by inhibiting pyroptosis, with its lung-protective effects partially mediated through the NRF2/HO-1 pathway (Li et al., 2023). However, whether TMPZ protects against AP through pyroptosis inhibition remains unclear.

This study hypothesizes that TMPZ has protective effects against AP, potentially via suppression of pyroptosis through the NRF2/HO-1 pathway. This study aims to elucidate the role of TMPZ in regulating pyroptosis and mitigating pancreatic tissue damage and inflammation in AP. Additionally, the study investigates the involvement of the NRF2/HO-1 signaling pathway in NLRP3-mediated pyroptosis, providing new insights into potential therapeutic strategies for AP.

## 2 Materials and methods

### 2.1 AP animal model

**FIGURE 1:**
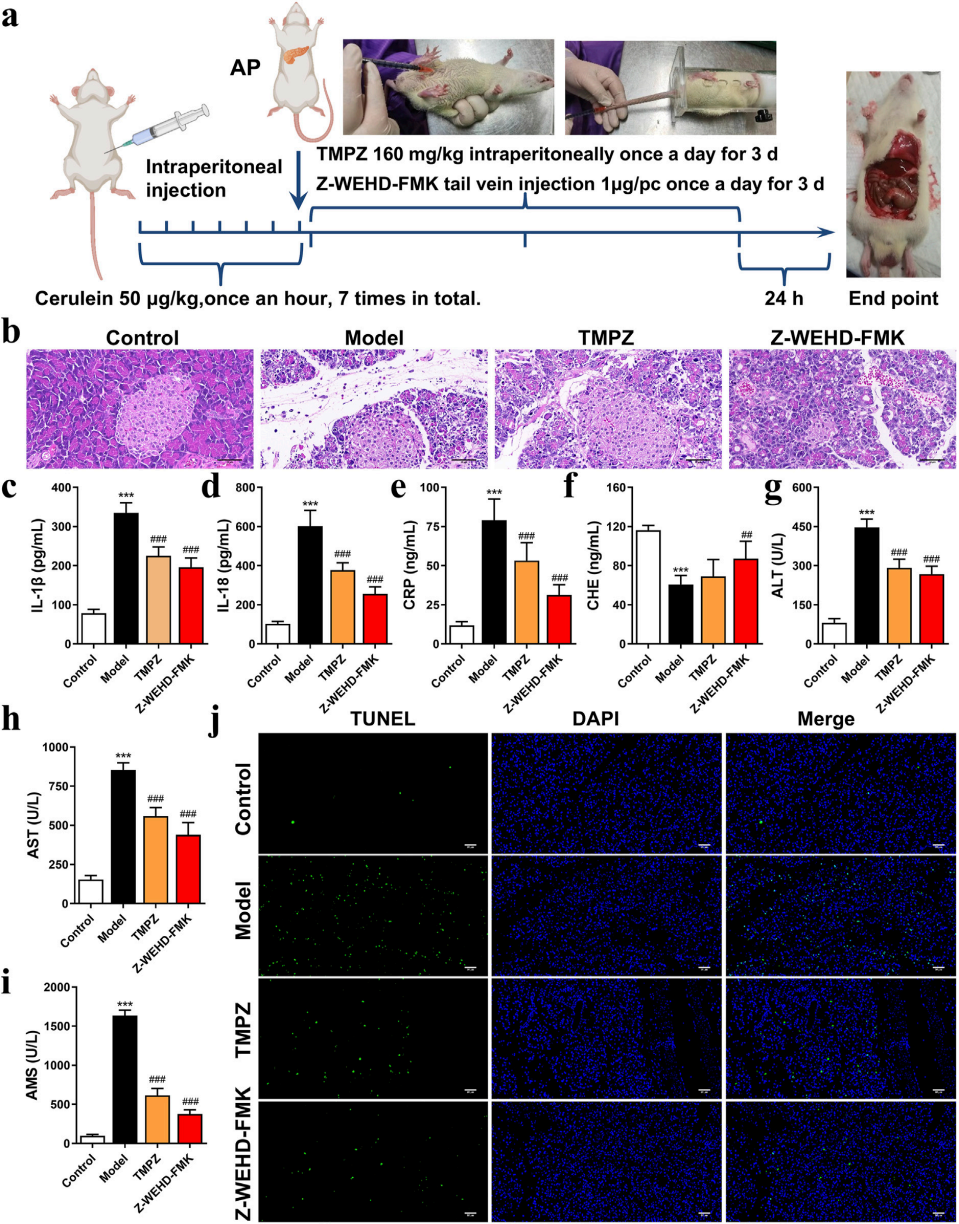
TMPZ attenuates inflammation in cerulein-induced AP rats **(a)** Experimental workflow for AP model establishment and drug administration. **(b)** Histopathological analysis of pancreatic tissue using HE staining (scale bar: 50 μm). **(c–i)** Serum levels of IL-1β, IL-18, CRP, CHE, AST, ALT, and AMS measured via ELISA and biochemical assays. Compared with the control group, ^***^P < 0.001; compared with the model group, ^##^P < 0.01, ^###^P < 0.001. **(j)** TUNEL staining for detecting cell apoptosis (scale bar: 50 μm) (n = 8 per group).

The drugs and reagents for the experiment were presented in Table 1. A total of 32 male Sprague-Dawley (SD) rats, aged 6–8 weeks and weighing 200–220 g, were procured from the Hubei Research Center of Laboratory Animals. The rats were housed under specific pathogen-free (SPF) conditions at a temperature of 25°C ± 1°C, humidity of 55% ± 5%, and a 12-h light/dark cycle, with free access to standard chow and water for 1 week prior to the experiment. Rats were randomly assigned into four groups (n = 8 per group): Control, AP model, TMPZ, and positive drug (Z-WEHD-FMK) groups. The AP model was induced via intraperitoneal injections of cerulein (50 μg/kg) at 1-h intervals for a total of seven injections (Elder et al., 2011). Rats in the Control group received equivalent volumes of saline intraperitoneally. In the TMPZ group, rats were treated with an intraperitoneal injection of TMPZ (160 mg/kg) 30 min after model induction, administered once daily for three consecutive days (Yang et al., 2005). The Z-WEHD-FMK group received a tail vein injection of Z-WEHD-FMK (1 μg/rat) 30 min post-induction, also administered once daily for three consecutive days (Figure 1a). All experimental protocols adhered to the guidelines of the Animal Care and Use Committee of the Hubei Provincial Center for Disease Control and Prevention (Approval No. 202420134, Date: 21 June 2024).

**TABLE 1:** The drugs and reagents for the experiment.

Drugs/reagents	Catalog numbers	Manufacturer
Cerulein	BD119547	Bidepharm (Shanghai, China)
Ham’s F-12K	BASmed-AW-00	ANwei-sci (Shanghai, China)
Cell counting kit-8	CK-04	Dojindo (Kyushu Island, Japan)
IL-1β ELISA Kit	E-EL-R0012c	Elabscience (Wuhan, China)
IL-18 ELISA Kit	E-EL-R0567c	Elabscience (Wuhan, China)
CRP ELISA Kit	ml038253	Enzyme-linked Biotechnology (Shanghai, China)
CHE ELISA Kit	ml037155	Enzyme-linked Biotechnology (Shanghai, China)
LDH Assay Kit	A020-2	Nanjing Jianjian Bioengineering Institute (Nanjing, China)
TMPZ	H12020888	Tianjin Jianyao Pharmaceuticals (Tianjin, China)
Z-WEHD-FMK	A1924	APExBIO (Houston, United States)
Hoechst-PI Pyroptosis Kit	40744ES6	Yesen Biotechnology (Shanghai, China)
TsingZol Total RNA Extraction Reagent	TSP401	Tsingke Biotechnology (Beijing, China)
SynScript™ III cDNA Synthesis Mix	TSK3225	Tsingke Biotechnology (Beijing, China)
2×TSINGKE^®^ Master qPCR Mix (SYBR Green I)	TSK201	Tsingke Biotechnology (Beijing, China)
dNTPs Mix (10 mM each)	TSK2200	Tsingke Biotechnology (Beijing, China)
jetPRIME Transfection Reagent	PT-114-75	Polyplus (Gottingen, Germany)
Double Antibiotic Solution	SV30010	Hyclone (Logan, United States)
T-PER™ Protein Extraction Kit	78,510	Thermo Fisher Scientific (Waltham, United States)
ASC antibody	bs-6741R	Bioss (Beijing, China)
GSDMD antibody	66387-1-Ig	Proteintech (Wuhan, China)
Caspase-1 antibody	22915-1-AP	Proteintech (Wuhan, China)
NLRP3 antibody	19771-1-AP	Proteintech (Wuhan, China)
HO-1 antibody	10701-1-AP	Proteintech (Wuhan, China)
NRF2 antibody	80593-1-RR	Proteintech (Wuhan, China)
GAPDH antibody	60004-1-Ig	Proteintech (Wuhan, China)
Lamin B1 antibody	66095-1-Ig	Proteintech (Wuhan, China)

### 2.2 Collection of serum and pancreatic tissue

At the end of the experiment, rats were anesthetized by intraperitoneal injection of sodium pentobarbital at a dose of 200 mg/kg, and blood was collected from the abdominal aorta (Correia-Pinto et al., 2002). Whole blood samples were left at room temperature for 2 h, centrifuged at 3,000 rpm for 10 min, and the serum was stored at −80°C for further analysis. Serum levels of amylase (AMS), alanine transaminase (ALT), and aspartate transaminase (AST) were measured using an automated biochemical analyzer. ELISA kits were used to quantify the expression of C-reactive protein (CRP), cholinesterase (CHE), IL-1β, and IL-18 according to the manufacturer’s instructions.

The euthanasia was performed by exsanguination under deep anesthesia. Following euthanasia, pancreatic tissues were excised and rinsed 1–2 times in pre-chilled PBS. The tissue was divided into two parts: one part was fixed in 4% paraformaldehyde for histopathological analysis (e.g., H&E staining, TUNEL assay, and immunofluorescence staining for NLRP3, GSDMD, and Caspase-1), while the other was snap-frozen for subsequent Western blotting and RT-qPCR.

### 2.3 Hematoxylin and eosin (H&E) staining

Pancreatic tissues were fixed in 4% paraformaldehyde for 48 h, dehydrated, embedded in paraffin, and sectioned at 5 μm thickness. The sections were deparaffinized and stained with H&E following the kit instructions, then examined under a light microscope.

### 2.4 TUNEL assay

Paraffin sections were deparaffinized with xylene and rehydrated through graded ethanol. After protease K treatment and PBS washing, TUNEL reagent was applied, and sections were incubated in a humidified chamber at 37°C for 2 h. DAPI was used to counterstain nuclei, followed by 10 min of incubation at room temperature in the dark. The slides were sealed with an anti-fluorescence quenching mounting medium and observed under a fluorescence microscope.

### 2.5 Transmission electron microscopy (TEM)

Fresh pancreatic tissues were diced into 1 mm^3^ cubes within 1–3 min and fixed in an electron microscopy-grade fixative. After washing with 0.1 M phosphate buffer (pH 7.4), tissues underwent secondary fixation, graded alcohol dehydration, and acetone treatment. Samples were infiltrated with Epon 812 embedding medium and polymerized at 60°C for 48 h. Ultrathin sections (60–80 nm) were cut, stained with 2% uranyl acetate and 2.6% lead citrate, and examined using a transmission electron microscope.

### 2.6 Immunofluorescence for NLRP3, Caspase-1, and GSDMD

Paraffin sections were deparaffinized and subjected to antigen retrieval using EDTA buffer (pH 8.0). After blocking with serum, primary antibodies against NLRP3 (1: 100), GSDMD (1: 100), and Caspase-1 (1: 100) were applied, followed by overnight incubation at 4°C. Secondary goat anti-rabbit IgG antibodies (1: 100) were added and incubated at room temperature for 1 h. Sections were counterstained with DAPI and sealed. Fluorescence microscopy was used to visualize and photograph the expression of NLRP3, GSDMD, and Caspase-1.

### 2.7 Establishment of AR42J cell AP model

AR42J cells were obtained from Procell (Wuhan, China), and cultured in Ham’s F-12K supplemented with 20% FBS and 1% P/S at 37°C with 5% CO_2_. Log-phase AR42J cells were seeded at a density of 2 × 10^4^ cells/well in 96-well plates and incubated overnight at 37°C in a 5% CO_2_ incubator. Cells were treated with 0, 50, or 100 nM cerulein for 6, 12, and 24 h to determine the optimal concentration and exposure time for AP cell induction.

AR42J cells were randomly divided into four groups: Control group (AR42J cells cultured in normal serum medium), Model group (AR42J cells induced with 100 nM cerulein for 24 h), Model + TMPZ group (AR42J cells treated with 0.4 mg/mL TMPZ for 3 h before induction with 100 nM cerulein for 24 h), and Model + TMPZ + Z-WEHD-FMK group (AR42J cells treated with 50 μM Z-WEHD-FMK and 0.4 mg/mL TMPZ for 3 h before induction with 100 nM cerulein for 24 h).

### 2.8 NRF2-siRNA transfection

To further investigate whether TMPZ attenuates AP via NRF2, AR42J cells were transfected with control siRNA (5′UUCUCCGAAC GUGUCACGUTT 3′, 5′TACGUGACACG UUCGGAGAATT 3′), NRF2-siRNA1 (5′GGAUGAAGAG ACCGGAGAAUUTT 3′, 5′AAUUCUCCGG UCUCUUCAUCCTT 3′), NRF2-siRNA2 (5′CGAGUUACAGU GUCUUAAUACTT 3′, 5′GUAUUAAGA CACUGUAACUCGTT 3′), or NRF2-siRNA3 (5′GUAUUAAGACA CUGUAACUCGTT 3′, 5′GGAAGUCUUCAG CAUGUUACGTT 3′) for 48 h AR42J cells were seeded at a density of 1 × 10^6^ cells/well in 6-well plates and transfected using Lipofectamine 2000 (Invitrogen, Carlsbad, CA, United States) according to the manufacturer’s protocol when the cells reached 60%–90% confluence. After 48 h of transfection, the relative expression of NRF2 was measured by PCR to identify effective interference sequences. After selecting the effective NRF2 interference sequence, AR42J cells were randomly assigned to five groups: siNC (NC siRNA-transfected for 48 h), Model + siNC (NC siRNA-transfected for 48 h followed by 100 nM cerulein induction for 24 h), Model + TMPZ + siNC (NC siRNA -transfected for 48 h, treated with 0.4 mg/mL TMPZ for 3 h, and then induced with 100 nM cerulein for 24 h), siNRF2 (NRF2-siRNA2-transfected for 48 h), Model + siNRF2 (NRF2-siRNA2-transfected for 48 h followed by 100 nM cerulein induction for 24 h), and Model + TMPZ + siNRF2 (NRF2-siRNA2-transfected for 48 h, treated with 0.4 mg/mL TMPZ for 3 h, and then induced with 100 nM cerulein for 24 h).

### 2.9 Cell counting kit-8 (CCK-8) assay

Three replicate wells were selected from each experimental group, and 10 μL of CCK-8 solution was added to each well. The cells were incubated in a 5% CO_2_, 37°C incubator for 3 h. Absorbance values at 450 nm were measured using a microplate reader to calculate cell viability.

### 2.10 Lactate dehydrogenase (LDH) assay

For each group, 100 μL of cell supernatant was collected and processed according to the LDH assay kit instructions. Absorbance was measured at 450 nm with a microplate reader to calculate LDH activity in each group.

### 2.11 Hoechst 33342/PI double staining assay

Cells were seeded into 24-well plates and cultured overnight at 5% CO_2_ and 37°C. Then, 5 μL of Hoechst 33,342 and 5 μL of PI staining solution were added, mixed, and incubated on ice for 30 min. Fluorescence microscopy was used for imaging.

### 2.12 Quantitative real-time polymerase chain reaction (RT-qPCR)

Total RNA was extracted from rat tissue samples and AR42J cells and reverse-transcribed into cDNA. PCR reactions were conducted according to the kit protocol with the following reaction mixture: 2 μL cDNA, 10 μL SYBR Green Mix, 0.4 μL of each forward and reverse primer, and 6.8 μL sterile water. Primer sequences are listed in Table 2. GAPDH was used as the reference gene, and relative mRNA expression levels were calculated using the 2^−ΔΔCt^ formula based on Ct values obtained from the real-time PCR instrument.

**TABLE 2:** Primer pairs and primer sequence required for the experiment.

Gene name	Sequence (5′-3′)	NM access codes	Size
GAPDH	GTA​TGA​CTC​TAC​CCA​CGG​CA	NM_017008.4	162 bp
	GTA​TGA​CTC​TAC​CCA​CGG​CA		
NLRP3	TCT​GCG​TGT​TGT​CAG​GAT​CT	NM_001191642.1	178 bp
	ACA​GTG​AAG​TAA​GGC​CGG​AA		
Caspase-1	GGT​TCC​CTC​AAG​TTT​TGC​CC	NM_012762.3	165 bp
	GTC​AAC​ATC​AGC​TCC​GAC​TC		
GSDMD	TGG​CTC​TGA​ATG​GGA​TAT​TCT​T	NM_001400994.1	179 bp
	CTT​CTT​CCT​CAT​TGG​TTC​CAT​C		
ASC	GGA​CCC​CAT​AGA​CCT​CAC​TG	NM_012661.2	222 bp
	ATG​AGT​GCT​TGC​CTG​TGT​TG		
HO-1	TGA​AGC​ACA​TGA​AGC​ACG​AG	NM_012580.2	159 bp
	GAA​CGT​GGT​CAT​CGG​TAA​GC		
NRF2	GTG​ATC​TGT​CCC​TGT​GTA​AAG​C	NM_001399173.1	185 bp
	TAG​CTC​TTC​CAT​TTC​CGA​GTC​A		

### 2.13 Western blotting

Total protein was extracted from rat tissue samples and AR42J cells using the T-PER™ Protein Extraction Kit. Proteins were quantified, subjected to electrophoresis, and transferred to PVDF membranes. Membranes were blocked in 5% milk/TBST at room temperature for 1 h, incubated overnight with primary antibodies (ASC, 1: 1000; NLRP3, 1: 1000; GSDMD, 1: 5000; Caspase-1, 1: 2000; NRF2, 1: 1000; HO-1, 1: 1000) at 4°C, and then with secondary antibodies at room temperature for 2 h. Enhanced Luminol Reagent and Oxidizing Reagent were diluted with ddH_2_O, mixed, and applied to the membrane. The PVDF membrane was exposed to the chemiluminescent reagent for 2 min, and then analyzed using a gel imaging system. Protein band gray values were quantified, with GAPDH and Lamin B1 as internal references, to calculate relative protein expression.

### 2.14 Statistical analysis

Experimental data were expressed as mean ± standard deviation ( $\bar{x}$ ± SD). Statistical analyses were conducted using SPSS 25.0 software. For comparison of multiple groups, one-way ANOVA was used for data with normal distribution, and nonparametric rank-sum test was applied for non-normally distributed data. For comparisons between two groups, t-tests were used for data with equal variances, while Welch’s t-test was used for unequal variances. Statistical significance was defined as P < 0.05.

## 3 Results

### 3.1 TMPZ attenuates inflammation and preserves pancreatic and hepatic function in AP rats

An animal model of AP was established via intraperitoneal injection of cerulein in rats (Figure 1a). Histopathological analysis revealed significant interlobular septal edema, inflammatory cell infiltration, and disrupted pancreatic architecture in the model group (Figure 1b). Additionally, serum levels of IL-1β, IL-18, CRP, AMS, ALT, and AST were significantly elevated, whereas CHE levels were decreased (Figures 1c–i), consistent with the characteristics of AP, confirming the successful establishment of the disease model.

TMPZ and Z-WEHD-FMK demonstrated protective effects on pancreatic tissues in AP rats (Figure 1b), significantly reducing serum levels of IL-1β, IL-18, CRP, AMS, ALT, and AST, while enhancing CHE levels (Figures 1c–i). Furthermore, TMPZ and Z-WEHD-FMK significantly reduced TUNEL-positive staining induced by cerulein in AP rats (Figure 1j). These findings indicate that TMPZ mitigates inflammation, reduces DNA fragmentation associated with excessive cell damage, and preserves pancreatic and hepatic function in AP rats.

### 3.2 TMPZ inhibits pyroptosis in AP rats

**FIGURE 2:**
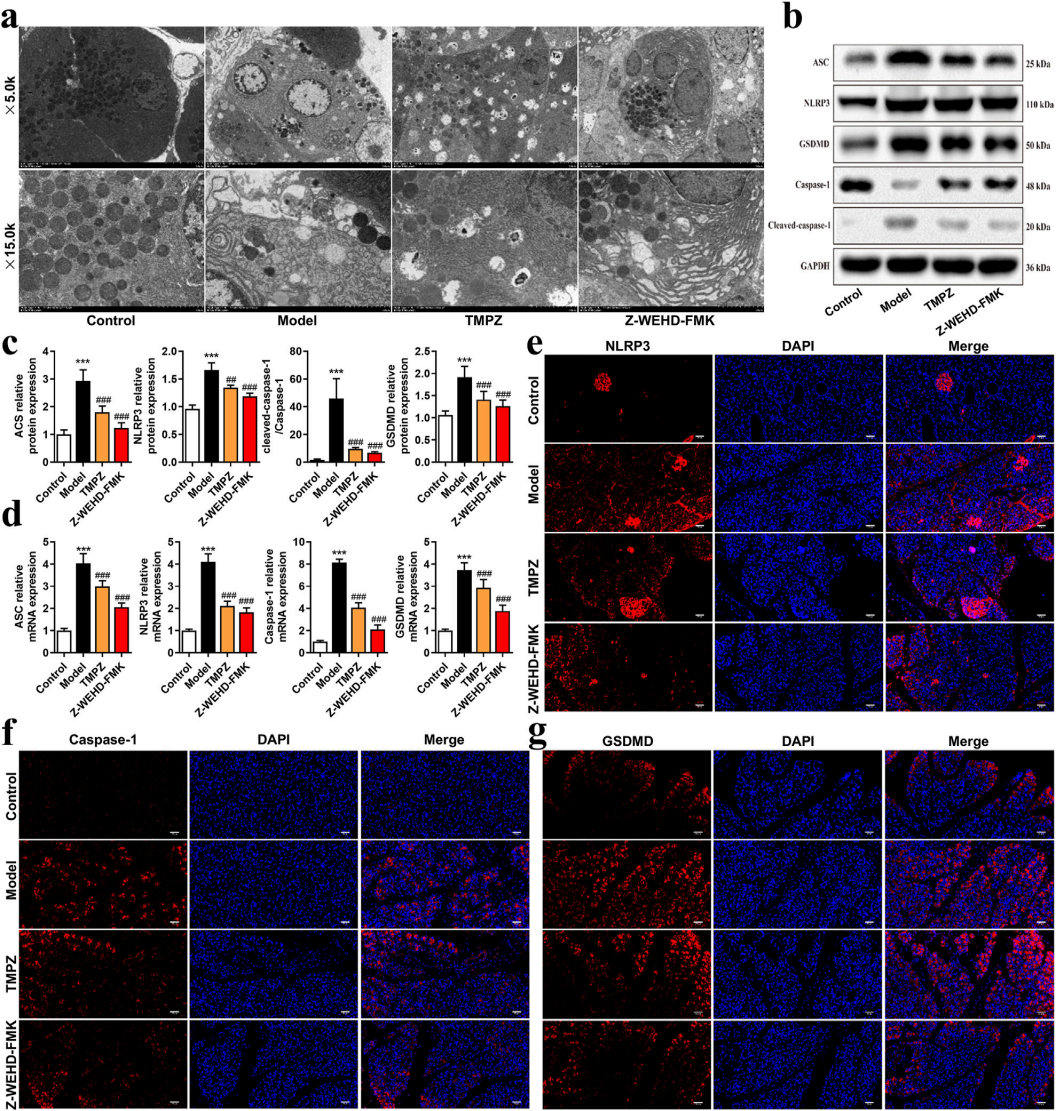
TMPZ inhibits pyroptosis in AP rat tissues **(a)** Transmission electron microscopy of pancreatic tissues to assess mitochondrial pyroptosis. **(b–d)** Western blot and RT-qPCR analysis of NLRP3, ASC, caspase-1, and GSDMD protein and mRNA levels. Compared with the control group, ^***^P < 0.001; compared with the model group, ^##^P < 0.01, ^###^P < 0.001. **(e–g)** Immunofluorescence analysis of NLRP3, caspase-1, and GSDMD expression (n = 8 per group).

The effects of TMPZ on pyroptosis in AP rat tissues were examined using transmission electron microscopy (Figure 2a). In the control group, cells displayed intact membranes, evenly distributed rough endoplasmic reticulum without dilation, mildly swollen mitochondria, and uniformly shaped zymogen granules. In contrast, cells in the model group exhibited features characteristic of pyroptosis, including partial membrane disruption, dilated and stressed endoplasmic reticulum, swollen mitochondria, and reduced, irregular zymogen granules. TMPZ and Z-WEHD-FMK treatment ameliorated cellular damage, with TMPZ-treated samples showing intact membranes and moderate mitochondrial damage, while Z-WEHD-FMK-treated samples displayed slight endoplasmic reticulum dilation and relatively preserved zymogen granules.

The NLRP3–ASC–caspase-1 protein complex is a classic inflammasome involved in mediating pyroptosis. Further analysis revealed that TMPZ significantly downregulated the expression levels of pyroptosis-associated proteins, including NLRP3, ASC, caspase-1, and GSDMD (Figures 2b–g, P < 0.01). These findings suggest that TMPZ inhibits pyroptosis and suppresses the activation of the NLRP3 inflammasome in AP rat tissues.

### 3.3 Inhibition of pyroptosis in acute pancreatitis cells by TMPZ

**FIGURE 3:**
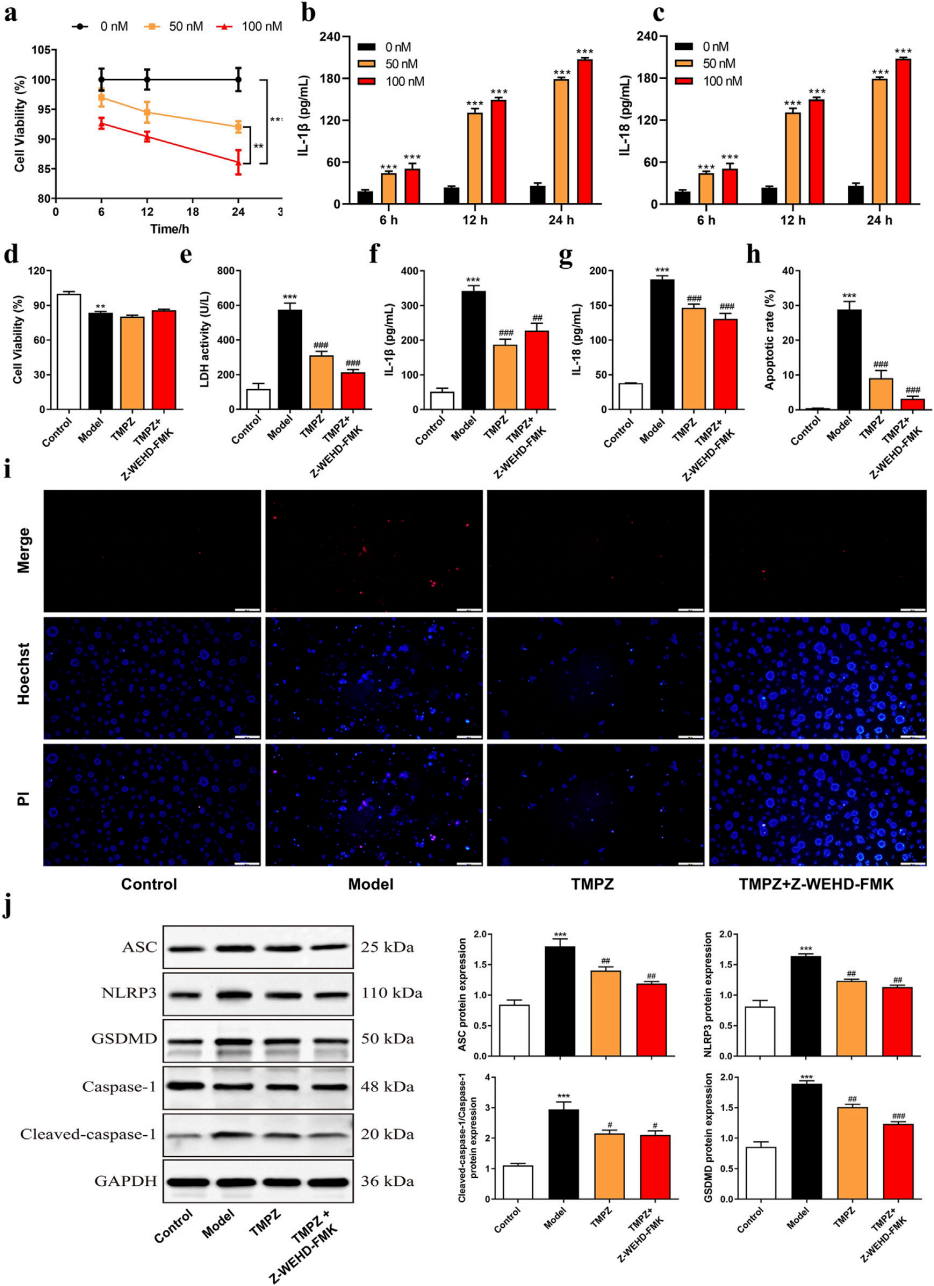
TMPZ suppresses pyroptosis in acute pancreatitis cells. **(a)** Cell viability and **(b,c)** levels of IL-1β and IL-18 in AR42J cells after stimulation with varying concentrations of cerulein (0, 50, and 100 nM) for 6, 12, and 24 h. Compared with the 0 nM group, ^**^P < 0.01, ^***^P < 0.001. Cells were divided into four groups: control (normal culture), model (cerulein treatment), TMPZ (pretreatment with TMPZ followed by cerulein treatment), and caspase-1 inhibitor fluoromethyl ketone (Z-WEHD-FMK, 50 μM; combined pretreatment with Z-WEHD-FMK and TMPZ followed by cerulein treatment). **(d)** Cell viability was detected by CCK-8 assay, **(e)** cytotoxicity by LDH assay, **(f,g)** levels of inflammatory cytokines IL-1β and IL-18 by ELISA, **(h,i)** apoptosis rate by Hoechst 33342/PI staining, and **(j)** protein expression levels of NLRP3, ASC, caspase-1, and GSDMD by Western blot. Compared with the control group, ^**^P < 0.01, ^***^P < 0.001; compared with the model group, ^###^P < 0.001. Three independent experiments performed in triplicate.

The cell viability of AR42J cells was assessed using a CCK-8 assay after stimulation with various concentrations of cerulein (0, 50, 100 nM) for 6, 12, and 24 h (Figure 3a). The results demonstrated a significant decrease in cell viability at a concentration of 100 nM cerulein after 12 and 24 h (P < 0.05), with cell viability declining to 86.10% at 24 h (P < 0.01). Additionally, ELISA was used to measure the levels of the inflammatory cytokines IL-1β and IL-18 induced by cerulein (Figures 3b,c). After 24 h of stimulation with 100 nM cerulein, IL-1β and IL-18 levels were significantly elevated (P < 0.001). Based on the CCK-8 and ELISA results, 100 nM cerulein for 24 h was selected for subsequent experiments to construct an *in vitro* AP cell model.

Cells were divided into Control, Model, Model + TMPZ, and Model + TMPZ + Z-WEHD-FMK groups, and cell viability and apoptosis were assessed using CCK-8, LDH, and Hoechst 33342/PI dual staining methods. The findings indicated that TMPZ did not significantly affect the viability of *in vitro* AP cells (Figure 3d) but significantly reduced cytotoxicity (LDH activity, P < 0.001), apoptosis rate (P < 0.001), and levels of inflammatory cytokines IL-1β and IL-18 (P < 0.01) in the model group (Figures 3e–i). There were no statistically significant differences in LDH, apoptosis rate, or levels of IL-1β and IL-18 between the Z-WEHD-FMK and TMPZ groups (Figures 3d–i), suggesting that TMPZ may reduce AP cell cytotoxicity, apoptosis, and inflammatory factor expression through a mechanism similar to that of Z-WEHD-FMK. Given the close association between caspase-1/5 and pyroptosis, we hypothesize that TMPZ may protect AP cells by inhibiting pyroptosis (Lu et al., 2023). Therefore, we used Western blotting to detect the effects of TMPZ on pyroptosis-related proteins (NLRP3, ASC, caspase-1, and GSDMD) in AP cells. The results showed that TMPZ significantly inhibited the expression levels of NLRP3, ASC, caspase-1, and GSDMD (Figure 3j, P < 0.01). The caspase-1/5 inhibitor Z-EHD-FMK did not significantly affect the expression of these proteins in TMPZ-treated AP cells, suggesting that TMPZ may inhibit pyroptosis via the caspase-1/5 pathway.

### 3.4 Detection of NRF2 pathway proteins and screening of NRF2 siRNA

**FIGURE 4:**
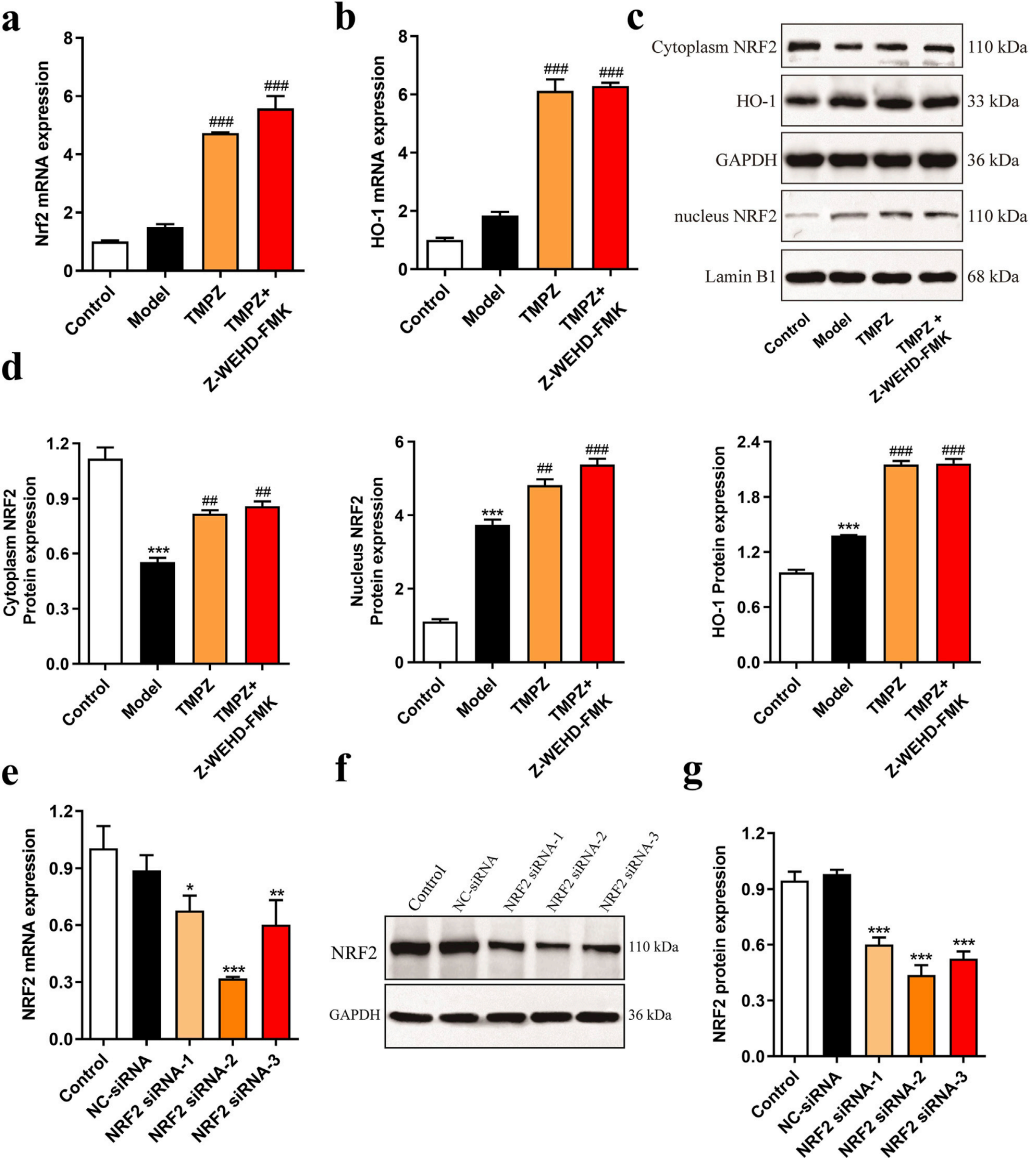
Detection of NRF2 pathway proteins and screening of NRF2 siRNA. **(a,b)** mRNA expression levels of NRF2 and HO-1 in each cell group as measured by RT-qPCR. **(c,d)** Protein expression levels of cytoplasmic NRF2, HO-1, and nuclear NRF2 by Western blot. Compared with the control group, ^***^P < 0.001; compared with the model group, ^##^P < 0.01, ^###^P < 0.001. **(e)** RT-qPCR analysis of NRF2 mRNA levels in AR42J cells transfected with NRF2 siRNA-1, -2, and -3. **(f,g)** Western blot analysis of NRF2 protein expression in AR42J cells transfected with NRF2 siRNA-1, -2, and -3. Compared with the NC siRNA group, ^*^P < 0.05, ^**^P < 0.01, ^***^P < 0.001. Three independent experiments performed in triplicate.

RT-qPCR analysis revealed that TMPZ significantly increased the mRNA expression levels of NRF2 and HO-1 in AP cells (Figures 4a,b, P < 0.001). Similarly, Western blot results showed a decrease in cytoplasmic NRF2 protein expression in the model group, while nuclear NRF2 and HO-1 protein levels increased compared to the control group (Figures 4c,d, P < 0.001). This indicates that during AP, cytoplasmic NRF2 translocates to the nucleus, increasing nuclear NRF2 expression, which subsequently initiates downstream HO-1 transcription and elevates HO-1 protein levels, consistent with changes observed in the NRF2/HO-1 signaling pathway during cellular oxidative stress (Gao et al., 2024). TMPZ significantly enhanced the expression of NRF2 and HO-1 in both the cytoplasm and nucleus compared to the model group (P < 0.01). No significant difference was observed in the NRF2/HO-1 pathway between the TMPZ and TMPZ + Z-WEHD-FMK groups, suggesting that this mechanism may involve the inhibition of caspase-1/5.

To further explore TMPZ’s mechanism of action, NRF2 siRNA-1/2/3 was transfected to assess its effects on NRF2 mRNA and protein expression in AR42J cells (Figures 4e–g). The results indicated that NRF2 siRNA-2 significantly reduced NRF2 mRNA and protein expression in AR42J cells (P < 0.001), making it the selected siRNA for subsequent experiments.

### 3.5 TMPZ inhibits pyroptosis of AP cells in vitro through the NRF2 pathway

**FIGURE 5:**
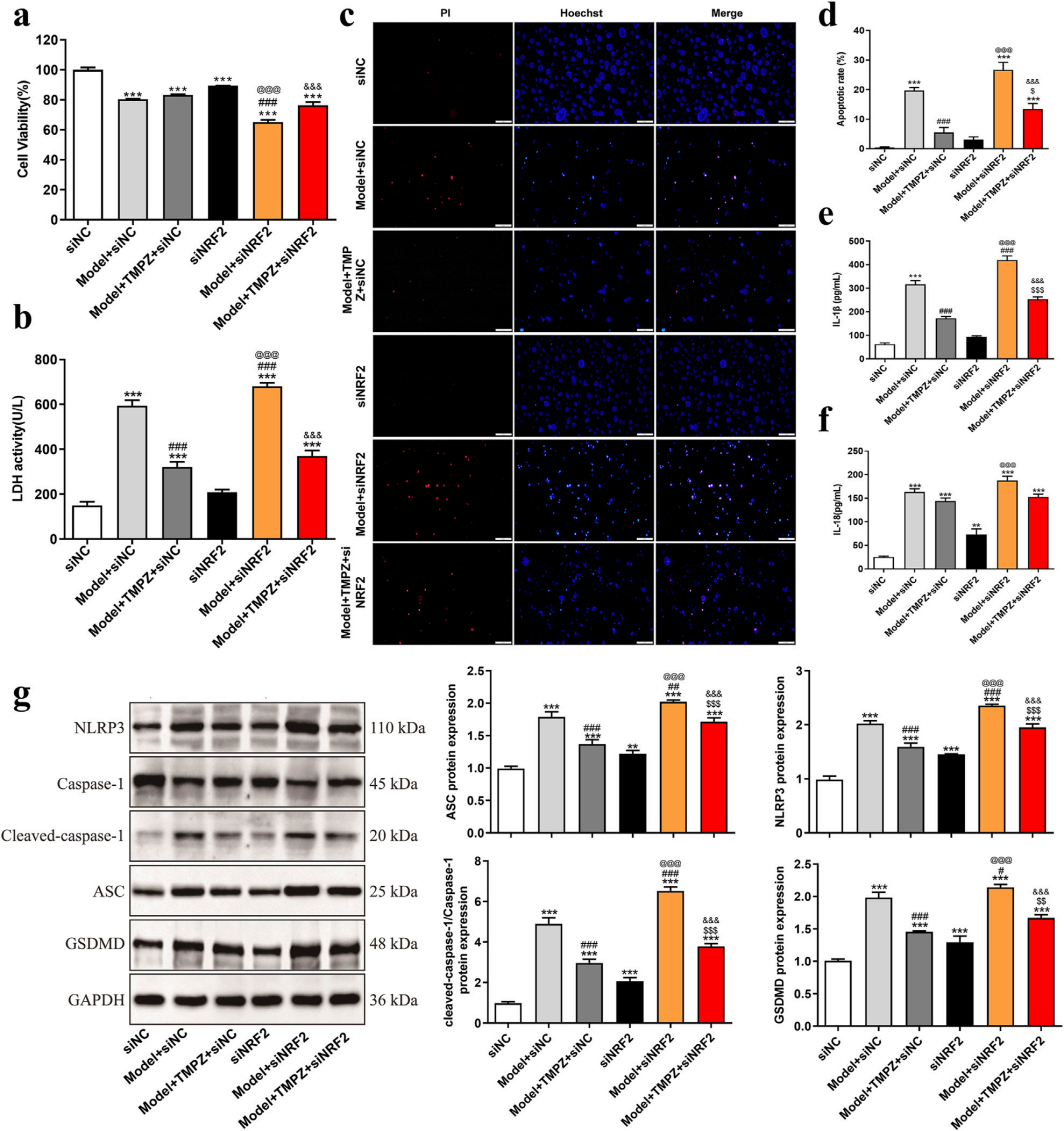
TMPZ inhibits pyroptosis in AP cells via the NRF2 pathway *in vitro*. Cells were divided into siNC, model + siNC, model + TMPZ + siNC, siNRF2, model + siNRF2, and model + TMPZ + siNRF2 groups. **(a)** Cell viability, **(b)** LDH activity, **(c,d)** apoptosis rate, **(e)** IL-1β levels, **(f)** IL-18 levels, and **(g)** protein expression levels of NLRP3, ASC, caspase-1, and GSDMD. Compared with the siNC group, ^**^P < 0.01, ^***^P < 0.001; compared with the model + siNC group, ^#^P < 0.05, ^###^P < 0.001; compared with the model + TMPZ + siNC group, ^$^P < 0.05, ^$$^P < 0.01, ^$$$^P < 0.001; compared with the siNRF2 group, ^@@@^P < 0.001; compared with the model + siNRF2 group, ^&&&^P < 0.001. Three independent experiments performed in triplicate.

Subsequently, the effects of TMPZ on cell viability, cytotoxicity, apoptosis rate, inflammatory cytokine levels, and pyroptosis-related gene expression in AP cells were examined following NRF2 siRNA transfection. Consistent with previous findings, the Model + TMPZ + siNC group showed increased cell viability compared to the Model + siNC group, though without statistical significance (Figure 4a). Conversely, LDH activity, apoptosis rate, and levels of inflammatory factors IL-1β and IL-18 were reduced (Figures 4b–f, P < 0.001). Compared with the model + siNRF2 group, the model + TMPZ + siNRF2 group showed increased survival rates, decreased LDH activity, apoptosis rates, and IL-1β levels (Figures 4a–f, P < 0.05). Compared to the Model + TMPZ + siNC group, TMPZ exhibited no significant impact on cell viability, LDH activity, or IL-18 levels in the Model + TMPZ + siNRF2 group. However, its inhibitory effect on apoptosis rate and IL-1β levels was diminished (Figures 5a–f, P < 0.05), suggesting that TMPZ may reduce apoptosis and IL-1β levels in AP cells through the NRF2 pathway.

Western blot analysis further demonstrated that, relative to the model + TMPZ + NC siRNA group, transfection with NRF2 siRNA attenuated the inhibitory effect of TMPZ on the expression of pyroptosis-related proteins NLRP3, ASC, caspase-1, and GSDMD in AP cells (Figure 5g, P < 0.001). These findings suggest that TMPZ inhibits the expression of pyroptosis-related proteins NLRP3, ASC, caspase-1, and GSDMD through the NRF2 pathway.

**FIGURE 6:**
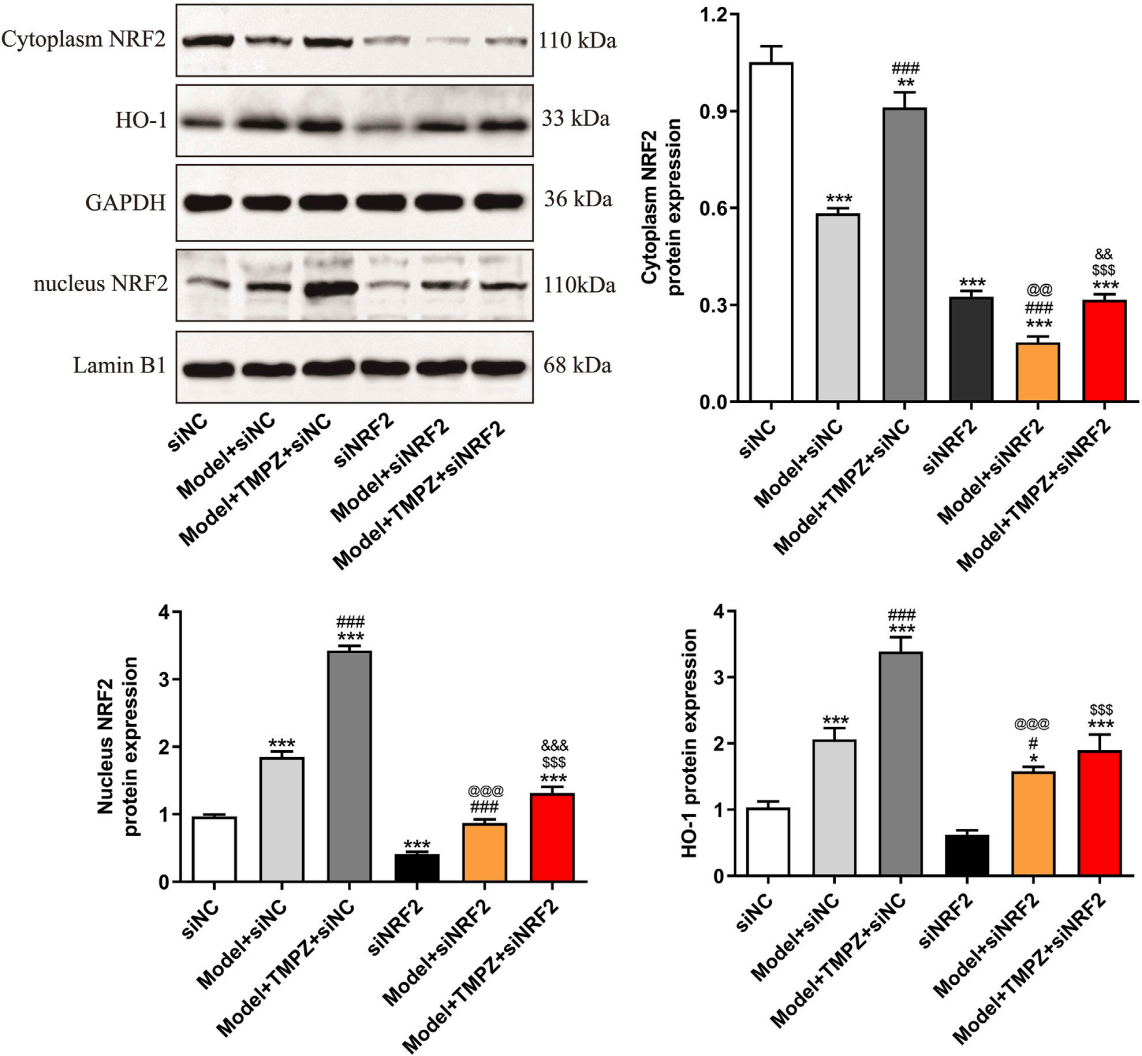
TMPZ increases NRF2 and HO-1 protein expression levels in AP cells via the NRF2 pathway *in vitro*. Compared with the siNC group, ^*^P < 0.05, ^***^P < 0.001; compared with the model + siNC group, ^#^P < 0.05, ^###^P < 0.001; compared with the model + TMPZ + siNC group, ^$$$^P < 0.001; compared with the siNRF2 group, ^@@^P < 0.01, ^@@@^P < 0.001; compared with the model + siNRF2 group, ^&&^P < 0.01, ^&&&^P < 0.001. Three independent experiments performed in triplicate.

Western blotting also examined NRF2/HO-1 pathway markers, specifically cytoplasmic and nuclear NRF2, as well as HO-1 protein levels (Figure 6). Results showed that TMPZ significantly upregulated Nrf2 and HO-1 protein expression in both the cytoplasm and nucleus of AP cells (P < 0.001), whereas NRF2 knockdown reduced the up-regulatory effect of TMPZ on Nrf2 and HO-1 protein expression (P < 0.001), confirming its action through the NRF2/HO-1 pathway.

## 4 Discussion

As an acute abdominal condition closely associated with inflammatory factors, AP can lead to both local and systemic complications, and in severe cases, can be life-threatening. In adjunctive therapy for AP, traditional Chinese medicine offers advantages such as low cost, broad pharmacological effects, minimal adverse reactions, and proven efficacy. TMPZ, a multifunctional compound extracted, isolated, and purified from the rhizome of natural medicinal plants, has demonstrated therapeutic potential in AP-related research (Chen et al., 2016; Chen et al., 2019). However, research on the specific mechanisms by which TMPZ protects against AP remains limited. This study demonstrates that TMPZ can alleviate pancreatic tissue damage, reduce inflammatory responses, and protect against liver damage in rats with AP. *In vitro* cell experiments further suggest that TMPZ may mitigate AP-induced inflammation and cell apoptosis by regulating the NRF2/HO-1 pathway. Moreover, TMPZ can inhibit the activation of the NLRP3 inflammasome and pyroptosis in AP cells, thereby exerting a protective role against AP.

The pathophysiology of AP is complex, leading to multiple cellular effects, including inflammatory responses. Studies have reported that IL-1β is a key pro-inflammatory cytokine triggering inflammation in AP (Gao et al., 2021; Liu et al., 2024; Niu et al., 2024), while IL-18 is considered an indicator of AP severity (Liu et al., 2024). Cerulein induces the production of a broad array of inflammatory mediators with high reproducibility, thereby facilitating the establishment of robust models for pancreatitis research (Sledzinski et al., 2013). Accordingly, this study utilized cerulein to construct *in vivo* and *in vitro* models of AP. Research indicates that cerulein induces inflammatory responses and promotes apoptosis (Wu et al., 2023; Zhou et al., 2023), aligning with our findings. Prior studies have found that TMPZ can reduce inflammation, mitigate the severity of AP, and protect against multiple organ damage in AP rats (Chen et al., 2019). Similarly, our findings demonstrate that TMPZ protects pancreatic and hepatic function, significantly reduces inflammatory cytokine levels induced by cerulein, suppresses apoptosis, and mitigates cytotoxicity in AP cells. The effect of TMPZ was not significantly impacted by Z-WEHD-FMK, suggesting that TMPZ’s protective effect on AP might be mediated by inhibiting caspase-1/5.

Caspases are a conserved family of cysteine proteases essential for programmed cell death and inflammation, and they mediate cell death through pyroptosis (Kesavardhana et al., 2020). Previous findings indicated that TMPZ improves lung injury by inhibiting apoptosis and pyroptosis (Jiang et al., 2021), but its effects on pyroptosis in AP cells have not been studied. Pyroptosis has been observed in acinar cells during AP (Al Mamun et al., 2022), and inhibiting pyroptosis has been shown to suppress apoptosis in AP cells and ameliorate pancreatitis (Liu et al., 2024). Thus, we propose that TMPZ may protect AP by inhibiting pyroptosis. Impaired pyroptosis-related signaling can activate the NLRP3 inflammasome, caspase-1, and GSDMD cleavage, accompanied by IL-1β and IL-18 maturation and release (Xue et al., 2019; Newton et al., 2024). In this study, TMPZ was shown to downregulate the expression of pyroptosis-related proteins (NLRP3, ASC, caspase-1, and GSDMD) in AP cells, suggesting that TMPZ inhibits pyroptosis in AP cells. Subsequently, we investigated the signaling pathway through which TMPZ inhibits cerulein-induced pyroptosis in AP cells.

As a critical endogenous antioxidant pathway, the NRF2 pathway is broadly present in various cells, including pancreatic cells, and has been shown to play a role in pyroptosis through the NRF2/HO-1 pathway (Wang et al., 2024b; Wang et al., 2024c). In this study, during AP, cytosolic NRF2 protein translocated to the nucleus, elevating nuclear NRF2 protein levels and initiating HO-1 transcription, which upregulated HO-1 protein expression. These results align with changes observed in the NRF2/HO-1 pathway in our previous studies on hypoxia/reoxygenation in cells (Gao et al., 2024). Our prior research indicated that activating the NRF2/HO-1 pathway inhibits hypoxia/reoxygenation-induced pyroptosis in H9C2 cardiomyocytes. Previous studies have demonstrated that TMPZ can mitigate damage from various diseases, such as acute lung injury in sepsis (Jiang et al., 2021; Li et al., 2023), myocardial ischemia-reperfusion injury (Wang K. et al., 2024), and coronary artery calcification, by inhibiting pyroptosis (Yang et al., 2024). Additionally, TMPZ’s protective effects are partly mediated via the NRF2/HO-1 pathway (Li et al., 2023; Hu et al., 2024). TMPZ has been reported to improve motor dysfunction through stimulation of the PGC-1/NRF2/HO-1 pathway (Wen et al., 2021). According to our findings, TMPZ pretreatment enhanced the expression of NRF2 and HO-1, and NRF2 knockdown partially reversed the protective effect of TMPZ pretreatment, confirming that TMPZ exerts its protective role in AP cells by inhibiting cerulein-induced pyroptosis via the NRF2/HO-1 pathway.

In conclusion, this study demonstrates that TMPZ effectively alleviates pancreatic and hepatic tissue damage, suppresses inflammatory responses, and inhibits apoptosis and pyroptosis in both *in vivo* and *in vitro* cerulein-induced AP models. These protective effects are at least partially mediated by the inhibition of NLRP3 inflammasome activation through the regulation of the NRF2/HO-1 pathway. These findings highlight the potential of TMPZ as a therapeutic agent for AP, suggesting that its beneficial effects may stem from modulating the NRF2/HO-1 pathway to inhibit pyroptosis and inflammation, offering a promising approach for the treatment of AP and other inflammatory conditions.

However, several limitations must be acknowledged. First, while our experimental design focused on validating the overall activation of the NRF2 pathway, it did not explore upstream regulatory mechanisms, such as the potential role of Keap1 in modulating NRF2 signaling. This critical aspect has been prioritized for further investigation in our further research. Second, the observation that TMPZ’s efficacy diminishes upon NRF2 siRNA knockdown confirms NRF2 as a necessary mediator but does not conclusively distinguish whether its regulation of GSDMD/NLRP3 is direct (e.g., via promoter binding) or indirect (e.g., through intermediate factors). Future studies incorporating chromatin immunoprecipitation (ChIP) assays or promoter-luciferase analyses will be essential to elucidate the precise mechanistic interactions. Additional studies are necessary to explore the broader clinical applicability of TMPZ and to clarify its precise mechanisms of action.

## Data Availability

The raw data supporting the conclusions of this article will be made available by the authors, without undue reservation.
